# Neuroprotective Effects of Thymol, a Dietary Monoterpene Against Dopaminergic Neurodegeneration in Rotenone-Induced Rat Model of Parkinson’s Disease

**DOI:** 10.3390/ijms20071538

**Published:** 2019-03-27

**Authors:** Hayate Javed, Sheikh Azimullah, MF Nagoor Meeran, Suraiya A Ansari, Shreesh Ojha

**Affiliations:** 1 Department of Anatomy, College of Medicine and Health Sciences, United Arab Emirates University, Al Ain, P.O. Box 17666, UAE; 2 Department of Pharmacology and Therapeutics, College of Medicine and Health Sciences, United Arab Emirates University, Al Ain, P.O. Box 17666, UAE; azim.sheikh@uaeu.ac.ae (S.A.); nagoormeeran1985@uaeu.ac.ae (M.F.N.M.); 3 Department of Biochemistry, College of Medicine and Health Sciences, United Arab Emirates University, Al Ain, P.O. Box 17666, UAE; sansari@uaeu.ac.ae

**Keywords:** neurodegeneration, oxidative injury, Parkinson’s disease, terpenes, rotenone, thymol

## Abstract

Parkinson’s disease (PD), a multifactorial movement disorder that involves progressive degeneration of the nigrostriatal system affecting the movement ability of the patient. Oxidative stress and neuroinflammation both are shown to be involved in the etiopathogenesis of PD. The aim of this study was to evaluate the therapeutic potential of thymol, a dietary monoterpene phenol in rotenone (ROT)-induced neurodegeneration in rats that precisely mimics PD in humans. Male Wistar rats were injected ROT at a dose of 2.5 mg/kg body weight for 4 weeks, to induce PD. Thymol was co-administered for 4 weeks at a dose of 50 mg/kg body weight, 30 min prior to ROT injection. The markers of dopaminergic neurodegeneration, oxidative stress and inflammation were estimated using biochemical assays, enzyme-linked immunosorbent assay, western blotting and immunocytochemistry. ROT challenge increased the oxidative stress markers, inflammatory enzymes and cytokines as well as caused significant damage to nigrostriatal dopaminergic system of the brain. Thymol treatment in ROT challenged rats appears to significantly attenuate dopaminergic neuronal loss, oxidative stress and inflammation. The present study showed protective effects of thymol in ROT-induced neurotoxicity and neurodegeneration mediated by preservation of endogenous antioxidant defense networks and attenuation of inflammatory mediators including cytokines and enzymes.

## 1. Introduction

Parkinson’s disease (PD) is pathologically described by the continued loss of dopaminergic neuronal cells in the substantia nigra pars compacta (SNc), which results in motor impairments such as loss of motion, postural and gait instability, resting tremors, and muscle rigidity [1,2]. Accumulating evidence suggests that mitochondrial dysfunction, lipid peroxidation, brain aging, and genetic susceptibility, which often involve oxidative stress and neuroinflammatory changes, play a major part in the pathogenesis of PD [3,4,5]. Oxidative stress and inflammation are the two central pathways in microglial cells activation that lead to progressive neuronal degeneration and represent an important therapeutic target in PD [3,4,5,6,7]. The activation and release of proinflammatory cytokines, such as IL-1β, IL-6, and TNFα, along with free radical generation including reactive oxygen species (ROS) and inducible nitric oxide synthase (iNOS), has detrimental effects on the existence of dopaminergic neurons in the SNc [6,7].

To ensure cellular homeostasis, a balance between pro- and antioxidant systems is typically required. Hence, the restoration of the cellular antioxidant system using antioxidants is one of the emerging therapeutic strategies to protect susceptible dopaminergic neurons from oxidative stress and subsequent inflammation. The adverse effects of anti-inflammatory agents and the pro-oxidant action of the synthetic antioxidants are of concern in therapeutics. This concern shifted the focus of drug discovery to explore plant extracts and plant-derived phytochemicals that possess antioxidant and anti-inflammatory activities for their therapeutic and preventive benefits in PD [8,9]. Therefore, in recent years, the focus of pharmacological therapy has been on the development of novel nutraceutical-based plant-derived phytochemicals that possess high antioxidant and anti-inflammatory properties, with a lesser degree of cytotoxic effects [9].

Among numerous plant-derived dietary phytochemicals, thymol has received attention due to its favorable physicochemical, pharmacokinetic, and pharmacological properties [10]. Thymol, a dietary monoterpene is chemically known as 2-isopropyl-5-methylphenol and found predominantly in many edible or culinary plants such as *Centipeda minima, Lippia multiflora, Nigella sativa, Ocimum gratissimum*, *Satureja hortensis*, *Satureja thymbra*, *Thymus spp. (Thymus vulgaris*, *Thymus pectinatus, Thymus zygis*, *and Thymus ciliates)*, *Trachyspermum ammi* and *Zataria multiflora* [10]. Thymol is catalogued as ‘Generally Recognized as Safe’ for use as a preservative and additive in food, beverages and cosmetic products, therefore it is considered to be safe for dietary use with minimal toxicity. Thymol exhibits potent pharmacological properties including antioxidant [11], anti-inflammatory [12], antimutagenic [13], analgesic [14], and anti-microbial [15] effects. It has been approved for use as a food additive and flavoring agent in cosmetics and food preparations. Its long-time dietary use, acceptable safety profile, and low toxicity have generated interest in evaluating its possible therapeutic use in neurodegenerative diseases. Therefore, in the current study we examined the neuroprotective efficacy and underlying mechanism of thymol in a rotenone (ROT)-induced rat model of neurodegeneration mimicking PD in humans. ROT, a plant-derived insecticide, inhibits mitochondrial complex I resulting in loss of ATP production, increase in oxidative stress, inflammation, prolonged glial cell activation, and nigrostriatal degeneration that mimics human PD [16,17,18,19]. The experimental models of ROT-induced neurodegeneration in rats, fruit fly or cell lines are popularly employed to screen and evaluate agents for their potential neuroprotective potential and therapeutic efficacy [20,21,22,23,24].

## 2. Results

### 2.1. Thymol Preserved TH+ Dopaminergic Neurons in SNc Regions and Dopaminergic Fibers in Striatum Regions of the Brain

**Figure 1:**
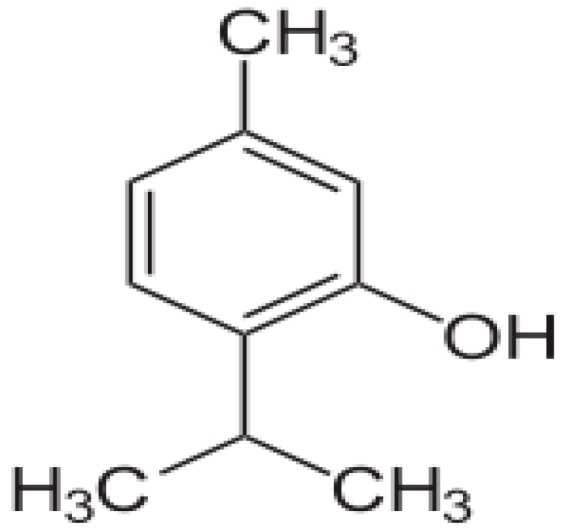
The chemical structure of thymol.

**Figure 2:**
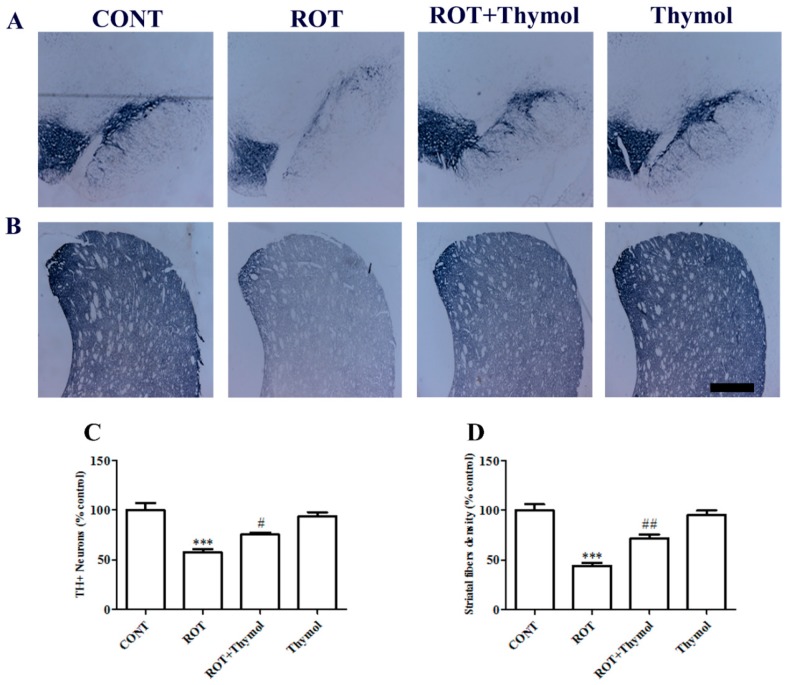
The illustrative photomicrograph showing expression of TH+ neurons in substantia nigra par compacta (SNc) (**A**) and TH-ir dopaminergic fibers in striatum (**B**). The scale bar is 100 µm. The expression of TH+ neurons and TH-ir fibers were reduced in the SNc region of rotenone (ROT) challenged rats as compared to vehicle injected rats in the CONT group. Thymol treatment to ROT challenged rats showed remarkable expressions of TH+ neurons and TH-ir fibers as compared to ROT injected rats. Quantification data showed significant (*** *p* < 0.001) decrease in the number of TH+ neurons and density of TH-ir fibers in ROT group rats compared to control rats. While thymol treatment to ROT injected rats showed significant (# *p* < 0.05; ## *p* < 0.01) increase in TH+ neurons and TH fibers density as compared to ROT alone injected rats (**C**,**D**).

In the current study, thymol (Figure 1), a monoterpene phenol was used to protect the dopaminergic neuronal death caused by ROT administration. We performed the immunohistochemical analysis of TH+ neurons in the SNc and TH-ir fibers in the striatum to observe the effects of thymol on nigrostriatal dopaminergic loss. The ROT injected animals showed significant (*p* < 0.001) degeneration of dopaminergic neurons in the SNc region when compared to rats of the control group received only vehicle (Figure 2A,C). Thymol administration significantly (*p* < 0.05) protected against ROT-induced degeneration of dopaminergic neurons. Dopaminergic neurons venture their axons to the striatum region wherein the terminal fibers are consisting of the dopamine transporter (DAT). Therefore, it was essential to examine whether the degeneration of dopaminergic neurons in the SNc region is associated with the loss of dopaminergic nerve terminals as evaluated by assessing the intensity of striatal TH-ir dopaminergic nerve terminal fibers. A significant (*p* < 0.001) loss in TH-ir fibers intensity was observed in animals challenged with ROT in comparison with animals of the control group received only vehicle. However, thymol pretreatment to ROT injected animals has produced a significant (*p* < 0.01) increase in the intensity of TH-ir nerve terminals compared to animals injected with ROT alone. This observation suggests the protective effect of thymol on dopaminergic neurons and nerve fibers (Figure 2B,D).

### 2.2. Thymol Inhibited Lipid Peroxidation and Restored GSH and Endogenous Enzymes Activity

**Figure 3:**
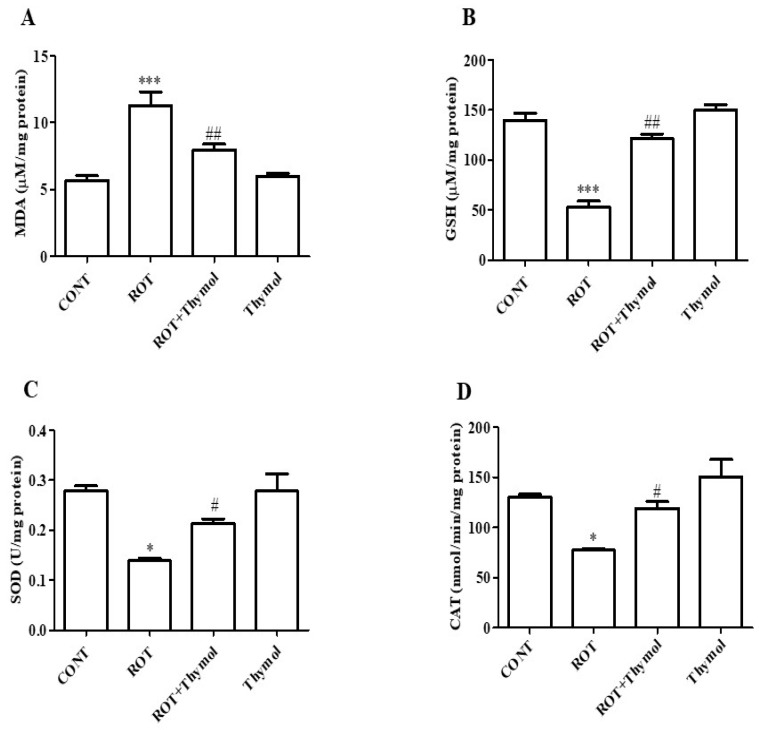
The levels of MDA, GSH and enzymatic activity of SOD and CAT were determined in the mid brain tissues of rats from different experimental groups. ROT treated rats showed significant (*** *p* < 0.01) increase in MDA (**A**) and decrease in GSH (**B**) levels when compared to control rats. Thymol treatment to ROT administered rats showed significantly (## *p* < 0.05) decreased level of MDA and increased (## *p* < 0.01) level of GSH. Moreover, ROT challenge also showed significant (* *p* < 0.05) decreased enzymatic activity of SOD (**C**) and CAT (**D**) when compared CONT rats. Thymol treatment to ROT challenged rats significantly (# *p* < 0.05) increased the activities of SOD and CAT when compared to ROT alone injected rats. The values are presented as mean ± SEM (*n* = 6–8).

The markers of lipid peroxidation, such as malondialdehyde (MDA), and the endogenous tripeptide antioxidant, glutathione (GSH), endogenous antioxidant enzymes (SOD and CAT) were measured in homogenates of the mid brain tissues. ROT administration induced a significant (*p* < 0.001, Figure 3A) rise in MDA levels in comparison with rats of control group. However, thymol treatment to the ROT challenged animals produced a significant (*p* < 0.01) decline in the MDA levels. ROT challenged rats show significant (*p* < 0.001) reduction in the levels of GSH as compared to control rats (Figure 3B). In contrast, thymol treatment significantly (*p* < 0.01) increase the GSH levels in ROT-injected rats compared to animals injected with ROT alone. Moreover, ROT injection also significantly decreases (*p* < 0.05) endogenous antioxidant enzyme activity such as*:* SOD and CAT in the ROT injected rats compared to control rats. However, thymol treatment significantly (*p* < 0.05) enhanced activity of SOD (Figure 3C) and CAT (Figure 3D) compared to ROT-injected animals. Further, thymol alone injected animals did not show any remarkable changes in the antioxidant enzymes activity.

### 2.3. Thymol Inhibited Activation of Glial Cells

**Figure 4:**
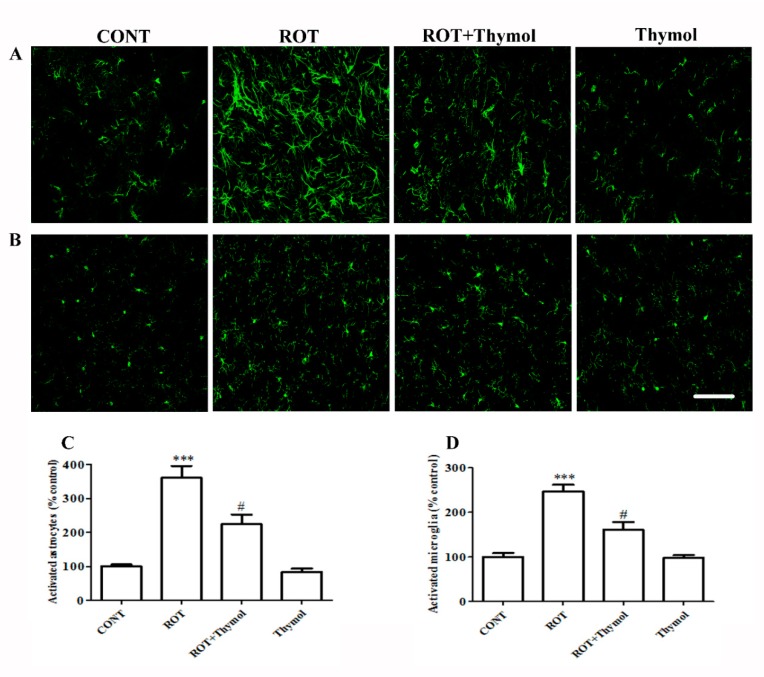
The immunofluorescence staining of GFAP (**A**) and Iba1 (**B**) in the striatum region of different experimental groups. Intense immunoreactivity of GFAP positive astrocytes (**A**) and Iba-1 positive microglia (**B**) were observed in the ROT challenged rats as compared to CONT rats. Thymol treatment to ROT challenged rats exhibited modest staining of GFAP and Iba-1 when compared to rats injected ROT (Scale bar 200 µm). Quantification data showed significant (*** *p* < 0.001) increased percentage number of activated astrocytes (**C**) and microglia (**D**) in ROT injected animals when compared to CONT rats. However, thymol treatment to ROT injected rats showed significantly (# *p* < 0.05) reduced percentage number of activated astrocytes and microglia as compared to rats injected with ROT alone. The values are presented as percent mean± SEM (*n* = 3).

Prolonged and sustained activation of the glial cells induces the release of inflammatory mediators including proinflammatory cytokines and inflammatory enzymes, which amplifies the neuroinflammatory process. We examined ROT-induced glial cells activation (astrocytes and microglia) in the striatum region. ROT injections significantly (*p* < 0.001) enhanced the expression of glial fibrillary acidic protein (GFAP) and ionized calcium binding adaptor protein (Iba-1) markers, which represent the number of activated astrocytes and microglial cells, respectively (Figure 4A–D). The increased expressions of GFAP and Iba-1 are considered the indices of inflammatory response following the activation of astrocytes and microglia. ROT administration caused a significant (*p* < 0.001) rise in the number of activated astrocytes and microglia as compared to rats received vehicle in control group. However, thymol treatment to ROT-administered rats led to a significant (*p* < 0.05) decrease in the quantity of activated astrocytes and microglial cells. Rats treatment with thymol alone did not exhibit notable activation of astrocytes and microglia when compared to the control animals, that is reasonable suggestive of its relative safety on astrocytes and microglia and aid in to the neuroprotective actions on the neurons.

### 2.4. Thymol Attenuated Activation of Proinflammatory Cytokines

**Figure 5:**
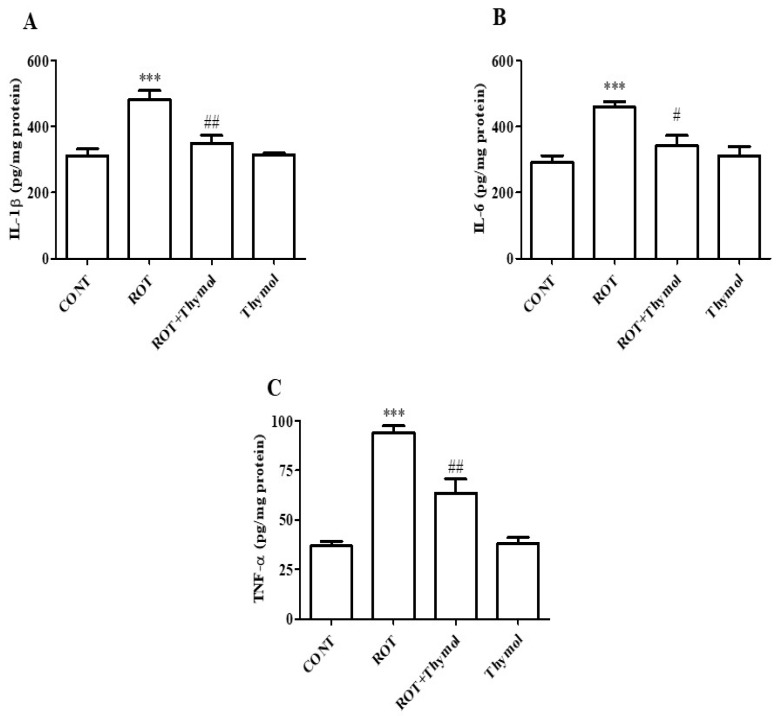
ELISA was used to quantify the level of proinflammatory cytokines; IL-1β, IL-6 and TNF-α in the mid brain tissues of rats from different experimental groups. The levels of IL-1β (**A**), IL-6 (**B**) and TNF-α (**C**) were significantly (*** *p* < 0.001) enhanced in ROT challenged rats when compared to CONT group rats. Thymol treatment to ROT challenged rats showed a significant (## *p* < 0.01; # *p* < 0.05) decrease in the levels of ROT-induced rise of proinflammatory cytokines. Additionally, the cytokines levels did no show significant difference in the rats of CONT and thymol alone groups. The values are presented as mean ± SEM (*n* = 6–8).

The increased secretion of inflammatory mediators, including proinflammatory cytokines, plays a key role in the etiopathogenesis and progression of PD. Therefore, the level of proinflammatory cytokines such as IL-1β, IL-6, and TNF-α, were quantified in ROT-challenged rats. A significant (*p* < 0.001) increase in the levels of IL-1β, IL-6, and TNF-α, were observed in ROT challenged rats compared to vehicle treated control rats (Figure 5A–C). However, thymol treatment to ROT injected rats significantly reduced the levels of IL-1β (*p* < 0.01), IL-6 (*p* < 0.05), and TNF-α (*p* < 0.01) compared to ROT alone injected animals (Figure 5A–C). The rats received thymol only did not cause substantial change in the level of proinflammatory cytokines compared to vehicle treated control animals.

### 2.5. Thymol Attenuated Expression Levels of COX-2 and iNOS

**Figure 6:**
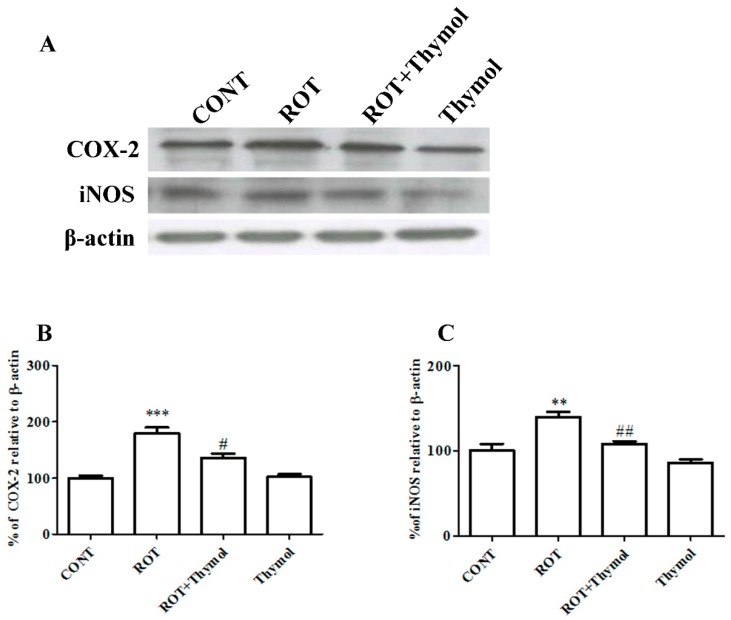
Striatal tissues were used to determine the expression levels of COX-2 and iNOS using western blotting (**A**). ROT challenge causes significant (*** *p* < 0.001) increase in COX-2 and iNOS levels when compared to CONT rats. Thymol treatment to ROT challenged rats exhibited significant (# *p* < 0.05; ## *p* < 0.01) decrease in the expression levels ofCOX-2 and iNOS as compared to rats received only ROT (**B**,**C**). Thymol alone treatment did not exhibit noteworthy change in the expression of COX-2 and iNOS when compared to vehicle injected rats of CONT group (*n* = 4).

We also examined the protein expression of inflammatory enzyme mediators such as COX-2 and iNOS by western blotting (Figure 6A–C). A significant (*p* < 0.001) rise in the expression of COX-2 and iNOS was observed in the striatal tissues of rats challenged with ROT in comparison with the vehicle injected rats in CONT group. Thymol treatment to ROT injected rats showed significantly reduced expression of COX-2 (*p* < 0.05) and iNOS (*p* < 0.01) when compared to rats challenged with ROT alone. However, the rats received thymol only was not found to produce significant alteration in the expression of COX-2 and iNOS compared to vehicle injected rats in CONT group. 

## 3. Discussion

The results of the present study demonstrate that thymol protect against ROT-induced neurodegeneration, mediating antioxidant and anti-inflammatory actions. The ROT model of neurodegeneration in rats is seemingly used as an experimental model for the assessment of agents for preventive and therapeutic efficacy and understanding the pathogenesis of PD [20,21]. The widespread activation of the microglia was observed in both the SNc and striatum following ROT challenge [18] and this appears consistent with the biochemical changes in the inflammatory mediators found in idiopathic PD [25,26], supporting the ROT model of PD. ROT induces nigrostriatal dopaminergic toxicity to mimic most of the pathological features of human PD including dopaminergic neurons loss, oxidative and nitrosative stress, impairment of the ubiquitin proteasome system and mitochondrial function along with α-synuclein aggregation and behavioral abnormalities [16,17,19].

Experimental and epidemiological studies suggest the health promoting properties and therapeutic benefits of numerous plant extracts, as well as their bioactive constituents, popularly known as phytochemicals, against various human diseases including PD [8,9,23,24]. Many phytochemicals have been found effective in treating numerous neurodegenerative diseases including PD [9,23,24,27]. Despite numerous pharmacological studies, there is no report available for the preventive or therapeutic potential of thymol against ROT induced neurodegeneration in in rats as an experimental model of PD. Additionally, thymol was found to inhibit β-amyloid (Aβ)-induced cognitive impairments in rats [28] that suggests thymol crosses the blood brain barrier and achieve the concentrations sufficient enough to exert its therapeutic effects on neurons. Therefore, in the current study we examined the neuroprotective role of thymol against ROT-induced neurodegeneration.

In the present study, a four-week regimen of ROT injections induced a significant degeneration of TH+ dopaminergic neurons in the SNc region and dopaminergic nerve fibers in the striatum of brain. TH+ neurons in the SNc region project their nerve terminals to the striatum. Therefore, the degeneration of dopaminergic neurons in the SNc area results in the diminution of dopaminergic nerve fibers/terminal in the striatum region. The loss of dopaminergic neurons and nerve terminals is reflected as one of the main pathological indices of PD. Importantly, thymol treatment protected the ROT-injected animals from the diminution of dopaminergic neurons and nerve terminals that is clearly suggestive of the neuroprotective effects of thymol against ROT-induced neurodegeneration.

ROT being highly lipophilic in nature easily crosses the blood brain barrier independent of any transporter and diffuses into neurons, accumulates in mitochondria and inhibits complex I. Mitochondrial complex I inhibition leads to loss of ATP production and subsequent rise in the ROS levels resulting oxidative stress [18,20,21]. Over generation of free radicals including ROS causes lipid peroxidation that is considered a crucial event in the etiopathogenesis of PD and an abnormal rise in the formation of MDA, a stable lipid peroxidation product, that has been shown in experimental and human studies [29,30]. Considerably, the brain tissues are highly susceptible to oxidative damage due to higher fatty acid contents, increased ROS level, and lessened endogenous enzymatic and non-enzymatic antioxidant defense components. We observed that thymol treatment significantly inhibited lipid peroxidation evidenced by reduced MDA levels in the midbrain tissues, which was induced by ROT injections and is suggestive of thymol’s lipid peroxidation inhibitory activity. The perturbation of endogenous non-enzymatic and enzymatic antioxidant defenses, such as GSH and SOD or CAT, has been well demonstrated in the brain tissues of experimental models and human PD [30]. The imbalance between the endogenous antioxidant defense system and ROS-induced oxidative stress is often linked with a simultaneous reduction in the GSH levels in the brain tissues with a concomitant fall in the activity of the intracellular antioxidant enzymes, SOD and CAT. To demonstrate the action of thymol on antioxidant defenses, we measured the activity of enzymatic antioxidants, SOD and CAT, and the level of non-enzymatic antioxidants, GSH. The administration of ROT induced a significant depletion of the levels of GSH and reduction in the activities of antioxidant enzymes SOD and CAT, whereas thymol treatment significantly restored the activity of antioxidant enzymes evidenced by improved antioxidant activity and prevented the depletion of GSH. This is suggestive of that thymol mitigates ROT-induced oxidative damage in brains attributed to its potent antioxidant and free radical scavenging properties. The reason for potent antioxidant and free radical scavenging property of thymol is ascribed to the presence of a phenolic hydroxyl group in its chemical structure that is believed to accountable for absorbing or neutralizing free radicals and augmenting endogenous antioxidants in protection against the deleterious effects of free radicals [31].

Chronic low grade sustained neuroinflammation is a contributing element of many neurodegenerative diseases including PD [4]. Neuroinflammation involves the activation of glial cells and secretion of classic inflammatory mediators such as proinflammatory cytokines and inflammatory enzymes; COX-2 and iNOS [32]. Given the crucial role of neuroinflammation in the onset and progression of PD, numerous studies so far have demonstrated the potential usefulness of anti-inflammatory drugs to decrease the development of neurodegeneration and lessen the risk factors for the individuals developing PD [33,34]. Though, the potential adverse effects of anti-inflammatory drugs limit their therapeutic use. Thymol has been shown to reduce inflammation by mitigating the onset and progression of the inflammatory processes in different experimental models of human diseases and appear safe in terms of adverse effects [35,36,37].

Therefore, we measured the levels of proinflammatory cytokines (IL-1β, IL-6, and TNF-α) in brain tissues of rats challenged with ROT. We observed that thymol treatment significantly reduced the release and activation of proinflammatory cytokines as evidenced by reduced levels in brain tissues of the rats challenged with ROT. Elevation in the activity and secretions of the proinflammatory cytokines, TNF-α, IL-1β, and IL-6 showed to participate in dopaminergic neurotoxicity and amplify the deleterious cascade of neurodegeneration in PD [38]. We also observed that thymol treatment significantly decreased the number of activated astrocytes and microglia in the striatum region in ROT-injected animals. The reduction in the number of glial cells following thymol treatment in ROT challenged rats is suggestive of its anti-inflammatory effects. Additionally, we also measured the expression of inflammatory enzymes mediators such as iNOS and COX-2, which rises following the induction of proinflammatory cytokines and increase in NF-κB, a transcription factor, in PD brains [39]. The COX-2 enzyme, an important physiologic and constitutive component of arachidonic acid metabolism pathway leads to the oxidation of dopamine to form dopamine-quinone conjugate that react with cysteinyl residues in proteins causes the alterations in protein structure and function [40]. These alterations further result in to neuronal cell death and suggested to be one of the probable explanations for the protective effect of COX-2 ablation [41].

Furthermore, activated glial cells, which express iNOS, are believed to enhance the levels of nitric oxide (NO) [5,42]. NO causes inhibition of the activity of several enzymes of the mitochondrial electron transport chain and leads the augmented generation of ROS. The crucial role of NO in PD pathogenesis is convincingly demonstrated in immunohistochemical studies performed on postmortem brain tissues that displays enhanced expression of iNOS in basal ganglia structures [43]. The current study findings shows that ROT injections elicited a remarkable increase in the expression of COX-2 and iNOS in the striatum, compared to control animals. However, the animals that received thymol treatment exhibited reduced expression of COX-2 and iNOS that is clearly suggestive of the potent anti-inflammatory effects of thymol.

## 4. Materials and Methods

### 4.1. Drugs and Chemicals

The antibodies used in this study included polyclonal rabbit anti-tyrosine hydroxylase (Novus Biologicals, Littleton, CO, USA), polyclonal rabbit anti-inducible nitric oxide synthase (iNOS), anti-cyclooxygenase-2 (COX-2), and anti-glial fibrillary acidic protein (GFAP) (Abcam, Cambridge, MA, USA), polyclonal rabbit anti-ionized calcium binding adaptor molecule-1 (Iba-1) (Wako Chemicals, Richmond, VA, USA), biotinylated secondary anti-rabbit antibody (Jackson Immunoresearch, West Grove, PA, USA), and Alexa fluor 488-conjugated goat anti-rabbit secondary antibodies (Life Technologies, Grand Island, NY, USA). The test compound, thymol was procured from Santa Cruz Biotechnology Inc, CA, USA. ROT, the chemical to induce PD in rats were purchased from Sigma Aldrich, St. Louis, MO, USA. The ELISA assay kits for antioxidant enzymes and glutathione (GSH) as well as other analytical grade reagents were also obtained from Sigma Aldrich, St. Louis, MO, USA.

### 4.2. Experimental Animals

The animal experiments were performed on five to six months old male adult albino Wistar rats weighing between 280–300 g. All the animals used in this study were provided by the animal research facility of College of Medicine and Health Sciences, United Arab Emirates University, Al Ain, United Arab Emirates. The animals were housed in polyacrylic cages under standard experimental animal housing conditions. The animals were maintained on a 12 h light/dark cycle and food and water was fed ad libitum. The animal experiments were performed following the guidelines and approval of Animal Ethics Committee of United Arab Emirates University, United Arab Emirates (ERA_2017_5500).

### 4.3. Experimental Design

In order to induce PD in rats, ROT was injected intraperitoneally once daily for 4 weeks with a dosage of 2.5 mg/kg body weight. The doses and schedule of ROT used for PD induction in rats in the present study was similar to that previously described and published report with slight modifications [22,23,24]. Briefly, a stock solution of 50× ROT was prepared in dimethyl sulfoxide and was used at a concentration of 2.5 mg/mL after dilution of stock in sunflower oil as vehicle. Thymol was prepared after dilution in sunflower oil at a concentration of 50 mg/2mL. The dose of thymol was selected based on previous studies [44,45] and was used at 50 mg/kg body weight through intraperitoneal injection 30 min prior to ROT challenge, once in a daily for a total of 4 weeks. The animals injected with same amount of oil only (vehicle) were designated as controls.

The animals were grouped in the following four categories as independent experimental groups of eight rats each. Group I: rats received vehicle injections, designated as normal control group (CONT). Group II: rats received rotenone and vehicle injections, designated as ROT group (ROT). Group III: rats received thymol 30 min prior to rotenone and vehicle injections, designated as thymol-treated group (ROT + Thymol). Group IV: rats received thymol injections alone, designated as thymol group (Thymol).

### 4.4. Tissue Collection

Animals of all the experimental groups were euthanized 48 h after the final administration of thymol or ROT to ensure a sufficient washout period. Prior to their sacrifice, animals received intraperitoneal injections of anesthesia pentobarbital (40 mg/kg body weight) followed by cardiac perfusion using phosphate-buffered saline (0.01 M, pH 7.4) to wash out the blood. Following perfusion, the brain was removed quickly, and the two hemispheres were separated. The midbrain and the striatum region were dissected out on ice from one of the hemisphere and the tissue was snap frozen under liquid nitrogen until further use. The other hemisphere was fixed with 4% paraformaldehyde solution for 48 h and subsequently exchanged with 10% sucrose solution three times a day for three consecutive days at 4 °C prior to cryostat sectioning.

### 4.5. Sample Preparation for Biochemical Studies

Tissue samples (mid brain) were prepared after lysis of the frozen midbrain tissues in KCL buffer supplemented with cocktail of protease and phosphatase inhibitor using a hand held tissue homogenizer separately for each group. The homogenate of each sample was centrifuged at 14,000 *g* for 20 min at 4 °C to get the post-mitochondrial supernatant for the quantification of endogenous enzymatic and no-enzymatic antioxidants, markers of lipid peroxidation, and levels of proinflammatory cytokines employing spectrophotometric assessment and enzyme-linked immunosorbent assay (ELISA).

### 4.6. Assessment of Lipid Peroxidation and Glutathione

The markers of lipid peroxidation, malondialdehyde (MDA) (North West Life science Vancouver, WA, USA) and glutathione (Sigma Aldrich, St. Louis, MO, USA), were estimated following the manufacturer’s protocol provided with the kit. The data are presented as µM/mg protein.

### 4.7. Assessment of Antioxidant Enzymes Activity

The activities of endogenous antioxidant enzymes such as superoxide dismutase (SOD) and catalase (CAT) were estimated following the protocols prescribed in manufacturer’s kits (Cayman Chemicals Company, Ann Arbor, MI, USA). The activities of SOD and CAT are expressed as U/mg protein, and nmol/min/mg protein, respectively.

### 4.8. Estimation of Proinflammatory Cytokines

ELISA assays were carried out in order to determine the quantity of proinflammatory cytokines such as interleukin-1β (IL-1β), interleukin-6 (IL-6), and tumor necrosis factor-alpha (TNF-α) in midbrain tissues, following the manufacturer’s protocol provided with the kits (R&D Systems, Minneapolis, MN, USA). The data are presented as pg/mg protein.

### 4.9. Immunohistochemistry of Tyrosine Hydroxylase (TH)

The immunohistochemical staining for TH was performed as published before [23,24]. Briefly, 14-μm thick coronal brain sections were sliced out at the level of the striatum and SNc using a cryostat (Leica, Wetzlar, Germany) and TH+ neurons in the SNc and TH-ir fibers in the striatum were evaluated following a method as described previously [23,24]. The loss of TH+ neurons in the SNc area after ROT administration was determined by enumerating the TH+ neurons at three different levels (section) (−4.8, −5.04, and −5.28 mm from the bregma) of the SNc region from each rat. A total three sections of each level and three rats per group were included in the analysis and the average count for each group is represented as a percentage. Therefore, in total, nine sections per group were analyzed for TH+ neurons. The differences in the optical density of TH-ir dopaminergic fibers in the striatum was measured using Image J software (NIH, Bethesda, MD, USA) in three different fields of each section (three sections/rat *n* = 3) with equal areas (adjacent to 0.3 mm from the bregma). An average of the three sections was calculated and is presented as a percentage compared to the control group. As background, the optical density was measured from the overlying cortex and the values obtained were subtracted from the values obtained for striatum. An investigator who was masked to the experimental groups and treatment was assigned to perform the enumeration of TH+ neurons and measurement of the optical density of the TH-ir fibers.

### 4.10. Immunofluorescence Staining of GFAP and Iba-1

Immunofluorescence microscopy was employed on 14-µm thick striatum sections to examine GFAP positive astrocytes and Iba-1 positive microglia using previously published protocols [23,24].

### 4.11. Determination of Activated Astrocytes and Microglia in the Striatum

In order to analyze the number of activated astrocytes and microglia, at least three coronal sections from a similar size of striatum from each animal and total three animals per group were utilized. The enumeration of activated astrocytes and microglia was undertaken based on the immunostaining intensity for GFAP and Iba-1 respectively and exhibiting morphological characteristics of hypertrophy and extended glial processes. The quantification of activated astrocytes and microglia were performed using Image J software (NIH, Bethesda, MD, USA) on the three randomly chosen equal area of different fields in each section.

### 4.12. Western Blot Analysis of COX-2 and iNOS

The tissues dissected from striatum of each experimental group were homogenized in 1X RIPA buffer supplemented with cocktail inhibitor of protease and phosphatase. The crude lysate was centrifuged at 14,000 rpm for 20 min in a refrigerated micro-centrifuge. A total of 35 μg of protein from each tissue sample was electrophoresed on a 10% SDS-polyacrylamide gel following a protocol as published before [23,24]. The blots were quantitated using image J software (NIH, Bethesda, USA).

### 4.13. Protein Estimation

The quantity of protein in samples were measured employing the Pierce BCA protein assay following the manufacturer’s instructions provided with the kit (Thermo Fisher Scientific, Rockford, IL, USA).

### 4.14. Statistical Analyses

The results are presented as the mean ± SEM. Statistical analysis were made using one-way analysis of variance (ANOVA) followed by Tukey’s test to calculate the statistical significance of differences between various groups including immunohistochemical cell/fiber count data. The data with *p*-values < 0.05 were considered significant.

## 5. Conclusions

Taken altogether, the present study clearly demonstrates that thymol provides protection against ROT-induced dopaminergic neurodegeneration, and the neuroprotective effects are attributed to the antioxidant and anti-inflammatory properties of thymol. Based on the findings of this study, it can be suggested that thymol or the herbs rich in thymol could be useful in the prevention of neurodegeneration in PD. Nonetheless, the translation of beneficial effects in humans and identification of the exact molecular mechanisms require further investigation.
